# Identification of new variants in patients with mucopolysaccharidosis in consanguineous Iranian families

**DOI:** 10.3389/fgene.2024.1343094

**Published:** 2024-02-15

**Authors:** Rezvan Zabihi, Mina Zamani, Majid Aminzadeh, Niloofar Chamanrou, Fatemeh Zahra Kiani, Tahere Seifi, Jawaher Zeighami, Tahere Yadegari, Alireza Sedaghat, Alihossein Saberi, Mohammad Hamid, Gholamreza Shariati, Hamid Galehdari

**Affiliations:** ^1^ Department of Biology, Faculty of Science, Shahid Chamran University of Ahvaz, Ahvaz, Iran; ^2^ Narges Medical Genetics and Prenatal Diagnosis Laboratory, Kianpars, Ahvaz, Iran; ^3^ Diabetes Research Center, Ahvaz Jundishapour University of Medical Sciences, Ahvaz, Iran; ^4^ Department of Genetics, Faculty of Science, Shahrekord University, Shahrekord, Iran; ^5^ Department of Medical Genetics, Faculty of Medicine, Ahvaz Jundishapur University of Medical Sciences, Ahvaz, Iran; ^6^ Cellular and Molecular Research Center, Ahvaz Jundishapur University of Medical Sciences, Ahvaz, Iran; ^7^ Department of Molecular Medicine, Biotechnology Research Center, Pasteur Institute of Iran, Tehran, Iran

**Keywords:** mucopolysaccharidosis (MPS), whole exome sequencing (WES), pathogenic variant, Sanger sequencing, genetic diagnosis

## Abstract

**Introduction:** Mucopolysaccharidoses are a group of lysosomal storage disorders that include seven types that are classified based on the enzymes that are disrupted. Malfunction of these enzymes leads to the accumulation of glycosaminoglycans (GAGs) in various tissues. Due to genetic and clinical heterogeneity, diagnosing and distinguishing the different types is challenging. Genetic methods such as whole exome sequencing (WES) and Sanger sequencing are accurate methods for detecting pathogenic variants in patients.

**Methods:** Thirty-two cases of mucopolysaccharidosis, predominantly from families with consanguineous marriages, were genetically examined. Out of these, fourteen cases underwent targeted sequencing, while the rest underwent WES. The results of WES were analyzed and the pathogenicity of the variants was examined using bioinformatics tools. In addition, a segregation analysis within families was carried out.

**Results:** In most cases, a pathogenic or likely pathogenic variant was detected. Sixteen previously reported variants and six new variants were detected in the known *IDS* (c.458G>C, c.701del, c.920T>G), *GNS* (c.1430A>T), *GALNS* (c.1218_1221dup), and *SGSH* (c.149T>C) genes. Furthermore, we discovered a c.259G>C substitution in the *NAGLU* gene for the first time in three homozygous patients. This substitution was previously reported as heterozygous. Except for the variants related to the *IDS* gene, which were hemizygous, all the other variants were homozygous.

**Discussion:** It appears that the high rate of consanguineous marriages in the families being studied has had a significant impact on the occurrence of this disease. Overall, these findings could expand the spectrum of pathogenic variants in mucopolysaccharidoses. Genetic methods, especially WES, are very accurate and can be used alone or in conjunction with other diagnostic methods for a more precise and rapid diagnosis of mucopolysaccharidoses. Additionally, they could be beneficial for family screening and disease prevention.

## 1 Introduction

Mucopolysaccharidoses refer to a group of metabolic disorders resulting from a deficiency of lysosomal enzymes involved in the breakdown of glycosaminoglycans (GAGs) and generally classified into seven types. The main cause of mucopolysaccharidosis type I (Hurler syndrome OMIM #607014, Scheie syndrome OMIM #607016, Hurler-Scheie syndrome OMIM #607015) is a mutation in the α-L-iduronidase (*IDUA*) gene, which results in a deficiency or relative deficiency of the α-L-iduronidase enzyme. This enzyme is responsible for the breakdown of dermatan sulfate and heparan sulfate (Hampe et al., 2020). In contrast to other MPS types with autosomal recessive inheritance, mucopolysaccharidosis type II (Hunter syndrome OMIM #309900) is an X-linked disease caused by a mutation in the iduronate-2-sulfatase (*IDS*) gene. The IDS protein triggers the initial cleavage of dermatan sulfate and heparan sulfate in the lysosome. Therefore, deficiency of this enzyme leads to the formation of these GAGs in various tissues (Verma et al., 2021). Mucopolysaccharidosis type III (Sanfilippo syndrome) is caused by a defect in one of four genes: N-sulfoglucosamine sulfohydrolase (*SGSH*) (MPS IIIA OMIM#252900), N-acetyl-alpha-glucosaminidase (*NAGLU*) (MPS IIIB OMIM#252920), heparan alpha-glucosaminide N-acetyltransferase (*HGSNAT*) (MPS IIIC OMIM#252930), and N-acetylglucosamine–Sulfohydrolase (*GNS*) (MPS IIID OMIM#252940). The protein products of these genes are involved in the lysosomal degradation of heparan sulfate (Yogalingam and Hopwood, 2001). Mucopolysaccharidosis type IV (Morquio syndrome) is determined by impairment of the enzymes galactosamine-6-sulfate sulfatase (GALNS) (MPS IVA OMIM#253000) or beta-galactosidase 1 (GLB1) (MPS IVB OMIM#253010), which are involved in the breakdown of keratan sulfate and chondroitin sulfate. Mucopolysaccharidosis type VI **(**Maroteaux-Lamy syndrome OMIM#253200**)** results from a deficiency of the enzyme arylsulfatase B (ARSB). This type begins with the accumulation of dermatan sulfate and chondroitin sulfate in the lysosomes. Finally, mucopolysaccharidosis type VII and type IX are caused by mutations in genes encoding the enzymes beta-glucuronidase (GUSB) and hyaluronoglucosaminidase-1 (HYAL1), respectively. These types are characterized by the accumulation of chondroitin sulfate, dermatan sulfate and heparan sulfate in MPS VII and hyaluronan in MPS IX (Celik et al., 2021; McBride and Flanigan, 2021; Nagral et al., 2022).

Overall, reduced activity of these enzymes leads to the accumulation of GAGs that affect various tissues, including the brain, eyes, ears, upper and lower respiratory tract, liver, spleen, heart, bones, cartilage, and joints, resulting in a variety of clinical manifestations (Münzer, 2011; McBride and Flanigan, 2021). The prevalence of each mucopolysaccharidosis subtype depends on geographic region and/or ethnic background. However, demographic data show that MPS is most common in Saudi Arabia, possibly due to consanguineous marriage or a founder effect. This is followed by Portugal, Brazil, the Netherlands and Australia (Celik et al., 2021). There is currently no curative treatment for mucopolysaccharidoses and treatment includes surgical, supportive and disease-specific treatments. Additionally, current standard treatments such as enzyme replacement therapy (ERT) and hematopoietic stem cell transplantation (HSCT) cannot prevent or reverse the abnormalities in the cornea, bones, central nervous system, and heart valves. In addition, ERT can trigger the formation of antibodies against the recombinant enzyme (Sawamoto et al., 2019). Therefore, early diagnosis of MPS is crucial to prevent numerous clinical symptoms. Since MPS syndromes are an extremely heterogeneous group of inherited diseases, the identification of new pathogenic variants could be of great benefit for family screening and prediction of the risk of recurrence. In addition, it can help with early diagnosis and provide therapeutic options for potential new cases. The aim of this study was to evaluate and validate potential novel variants involved in the pathogenesis of MPS using whole-exome sequencing and Sanger sequencing. We have reported several new variants in different types of genes associated with mucopolysaccharidosis and confirmed their association with the disease in the affected families.

## 2 Material and method

### 2.1 Patients

We enrolled 32 unrelated cases, including one neonatal death, who were referred to the Narges Medical Genetics and Prenatal Diagnosis laboratory in the Southwest of Iran from 2014 to 2022. These patients were given a preliminary diagnosis of Mucopolysaccharidosis by a metabolism specialist based on clinical symptoms and biochemical tests, such as cellular enzymatic activities and urine GAG levels. To accurately diagnose the type of disease and screen it in the patient’s families, we conducted genetic examinations. Genetic counseling was provided to all patients by genetic specialists from the laboratory. The study was approved by the Institutional Review Board of Ahvaz Jundishapur University of Medical Sciences, and informed consent was obtained from all enrolled patients and their families.

### 2.2 DNA extraction

Ten milliliters of peripheral blood were isolated from each case and their available respective parents. Genomic DNA was extracted using the standard salting-out protocol (Miler et al., 1988). The quality and quantity of the extracted DNA were evaluated using gel electrophoresis and a nanodrop, respectively.

### 2.3 Exome sequencing

Exome enrichment was performed on 18 samples using the SureSelect Human All Exon kit V6 (Agilent Technologies, Santa Clara, CA, United States), followed by sequencing using the Illumina HiSeq 2000 genome analyzer platform (Illumina, San Diego, CA, United States). The short reads were aligned to the human genome reference version B37 using BWA, and duplicate reads were marked using Picard v2.6.0 (https://broadinstitute.github.io/picard). GATK and ANNOVAR were used for variant detection and annotation, respectively.

### 2.4 Assessment of variant pathogenicity

To identify disease-causing variants, filtering was performed for each WES data file based on allele frequency. Variants with allele frequencies lower than 0.01 in the 1,000 Genomes (https://www.internationalgenome.org), ExAC (http://exac.broadinstitute.org), and gnomAD (https://gnomad.broadinstitute.org/) databases were selected for the next step. Then, the data files were filtered based on the variant consequence, selecting exonic, exonic-splicing, and splicing locations. Afterward, we focused on zygosity and the inheritance of diseases in families. Mucopolysaccharidosis-related genes were then filtered based on the OMIM and GeneCards databases. In the next step, the pathogenicity of the remaining variants was evaluated using *in silico* tools such as Mutation-Taster (Steinhaus et al., 2021), Polyphen2 (Adzhubei et al., 2010), PredictSNP2 (Bendl et al., 2016), VarSome (Kopanos et al., 2019), and CADD (Kircher et al., 2014). Finally, the selected variants were searched in our in-house database (which includes more than 2,500 WES files), and variants with low allele frequency were chosen for additional segregation analysis in the families.

### 2.5 Sanger sequencing

Direct Sanger sequencing was performed on DNA samples from 14 cases. Specific primers were designed using OLIGO 7 to target the entire coding exons and flanking intronic sequences of the *IDS, NAGLU, SGSH, ARSB, and GALNS* genes in order to identify any existing variants. The selected regions were amplified using PCR. The PCR products were then sequenced and analyzed using the ABI Prism 3700 automated genetic analyzer (Applied Biosystems, ThermoFisher, United States). The results were interpreted using Chromas 2.6.6 and compared with reference gene sequences using the BLASTN and ClustalW programs. Furthermore, the presence of all detected variants was confirmed through Sanger sequencing of cases that underwent whole exome sequencing, as well as available relatives of all the studied cases.

## 3 Result

**FIGURE 1:**
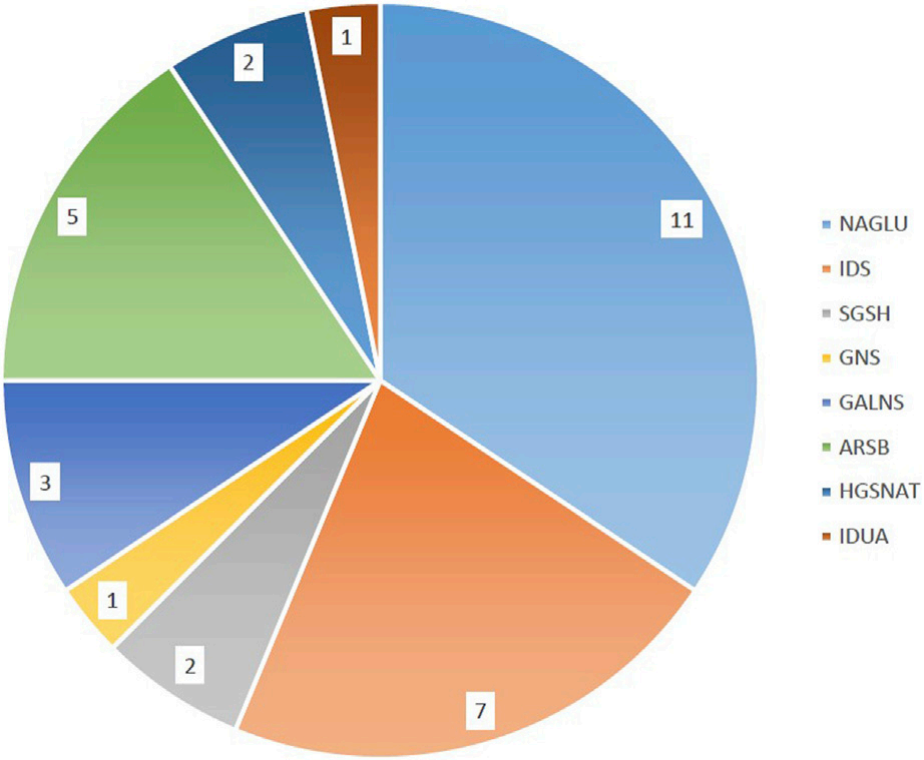
Distribution of variants in MPS genes. The majority of patients had disease-causing variants in the *NAGLU* and *IDS* genes respectively.

The initial diagnosis of mucopolysaccharidosis in patients was based on clinical symptoms and biochemical tests. However, the specific type of disease was not clear in some patients. After sequencing, the exact type of disease in each person was determined by identifying the presence of pathogenic or likely pathogenic variants in different known genes that encode enzymes responsible for mucopolysaccharidosis. The general information of the probands is provided in Table 1. Based on Table 1, the majority of patients were of Arab or Lor ethnicity, and 71.8% (23/32) of them were born to consanguineous parents. Furthermore, MPS III has the highest prevalence among all types of the disease, accounting for 50% (16/32) of cases. We identified various disease-causing variants in known genes associated with different types of Mucopolysaccharidosis in all 32 cases. The genetic findings and *in silico* analysis of these variants are summarized in Table 2. The distribution of genes containing these variants is shown in Figure 1, depicted as a pie chart. The *NAGLU, IDS, SGSH, GNS, GALNS, ARSB, HGSNAT*, and *IDUA* genes each contained one or more variants. Based on the data presented in Figure 1, it is evident that the *NAGLU* and *IDS* genes had the highest number of variants among the patients, respectively. Among all the variants, we detected 6 novel variants in the *IDS, GNS, GALNS*, and *SGSH* genes. All of these new variants occur in the protein-coding regions of the genes, with 66.6% (4/6) being missense mutations and 33.3% (2/6) being frameshift mutations. The novel variants are as follows, based on the gene in which the mutation occurred. Additionally, the pedigree of the proband’s families with new variants is shown in Figure 3.

**TABLE 1:** A brief description of individuals. P29 clinical data were not available due to neonatal death. P: proband, AR: autosomal recessive, NA: not available, Y:year, ND: neonatal death.

Clinical synopsis	P1	P2	P3	P4	P5	P6	P7	P8	P9	P10	P11	P12	P13	P14	P15	P16
OMIM Phenotype/inheritance	MPS Ih/s/AR	MPS II/XLR	MPS II/XLR	MPS II/XLR	MPS II/XLR	MPS II/XLR	MPS II/XLR	MPS II/XLR	MPS IIIA/AR	MPS IIIA/AR	MPS IIIB/AR	MPS IIIB/AR	MPS IIIB/AR	MPS IIIB/AR	MPS IIIB/AR	MPS IIIB/AR
Severe or mild Short stature	+	+	-	+	+	+	+	+	-	-	-	+	-	-	-	-
Macrocephaly	+	+	+	-	+	+	+	+	-	-	-	+	-	-	+	+
Coarse facial features	+	+	+	+	+	+	-	-	-	+	+	+	+	+	-	-
Hearing impairment	+	+	-	-	-	-	+	-	-	-	+	-	+	+	+	-
Otitis	-	-	-	-	-	-	-	-	+	-	-	-	-	-	-	-
Visual impairment	+	-	-	-	-	-	+	-	-	-	-	-	-	-	-	+
Hypertelorism	-	-	-	+	-	-	-	-	-	-	-	-	-	-	-	-
Synophrys	-	+	-	-	+	+	-	-	-	+	-	-	-	-	-	-
Macroglossia	+	-	+	+	+	+	+	+	-	-	-	+	-	-	-	-
Thick vermilion border	-	-	-	-	+	-	+	+	-	+	-	-	-	-	+	+
Delayed eruption of teeth/Widely spaced teeth	-	-	-	-	-	+	-	+	-	+	-	-	-	-	-	-
Short neck	-	+	+	-	+	+	+	+	-	+	-	+	-	-	-	-
Scoliosis	-	-	-	+	+	-	-	-	-	-	-	+	-	-	-	+
Joint stifness	+	-	+	-	+	+	+	+	-	-	+	-	-	-	-	+
Epidermal acanthosis	+	-	+	-	-	-	-	+	-	-	-	-	-	-	-	-
Hirsutism	+	+	-	+	+	+	-	+	+	+	+	+	+	+	-	-
Abnormality of the cardiovascular system	+	-	+	-	-	+	-	-	-	-	-	+	-	-	+	+
Respiratory problem	+	-	-	-	-	+	+	-	-	-	-	-	+	-	-	+
Hepatomegaly	+	+	+	-	+	+	+	+	-	-	-	-	-	+	-	-
Splenomegaly	+	+	+	-	+	+	+	+	-	-	-	-	-	-	-	-
Diarrhea	-	+	-	-	-	-	-	-	-	-	-	-	-	-	-	-
Inguinal hernia/Umbilical hernia	+	-	+	-	-	+	+	-	-	-	-	-	-	-	-	+
Kyphosis	-	+	-	-	-	-	-	-	-	-	-	-	-	-	-	-
Inability to walk	+	-	-	+	-	+	-	+	-	+	-	-	-	-	-	-
Ataxia	-	-	-	-	-	-	-	-	+	-	-	-	-	-	+	-
Loss of speech	-	+	-	+	-	+	-	+	+	+	+	-	+	+	+	+
Seizure	-	+	-	-	-	-	-	-	-	-	-	-	-	-	-	-
Intellectual disability	-	+	+	+	+	+	-	-	-	+	+	+	+	+	+	+
Sleep abnormality	-	-	-	-	+	-	-	+	+	-	-	-	-	-	-	+
Hoarse voice	-	+	-	+	-	+	-	-	-	-	-	-	-	-	-	-
Hyperactivity	-	-	+	-	-	-	-	-	+	+	-	-	-	-	+	+
Abnormal aggressive, impulsive or violent behavior	-	-	+	+	+	-	+	-	-	-	+	-	+	+	-	+
Sex	Female	Male	Male	Male	Male	Male	Male	Male	Female	Male	Female	Male	Female	Female	Male	Female
ethnicity	Lor	Lor	Arab	Lor	Lor	Lor	Lor	Lor	Lor	Persian	NA	Lor	Lor	Lor	Arab	Lor
consanguinity	+	+	-	+	-	-	-	+	+	+	NA	+	+	+	+	+
Age	8Y	7Y	7Y	15Y	12Y	13Y	11Y	10Y	8Y	14Y	NA	7Y	4Y	7Y	8Y	7Y

P17	P18	P19	P20	P21	P22	P23	P24	P25	P26	P27	P28	P29	P30	P31	P32
MPS IIIB/AR	MPS IIIB/AR	MPS IIIB/AR	MPS IIIB/AR	MPS IIIB/AR	MPS IIIC/AR	MPS IIIC/AR	MPS IIID/AR	MPS IVA/AR	MPS IVA/AR	MPS IVA/AR	MPS VI/AR	MPS VI/AR	MPS VI/AR	MPS VI/AR	MPS VI/AR
-	-	+	+	-	-	-	-	+	+	+	-	NA	+	+	+
+	+	+	+	-	+	-	-	-	+	-	+	NA	+	+	+
-	-	-	+	-	-	-	-	-	-	-	-	NA	-	+	-
+	-	+	-	-	-	+	-	-	+	+	-	NA	+	+	-
-	-	-	-	-	-	-	+	-	-	-	-	NA	-	-	-
-	-	+	-	-	+	-	-	-	+	-	+	NA	-	+	+
-	-	-	-	-	-	-	+	-	-	-	_	NA	-	-	-
+	-	-	-	-	-	-	-	-	-	-	-	NA	-	-	-
-	-	+	-	-	-	-	-	-	+	+	-	NA	+	+	+
+	-	-	-	-	-	+	-	-	-	-	-	NA	+	+	-
-	-	+	+	+	-	+	-	-	-	+	-	NA	+	+	-
+	+	+	-	-	-	+	-	-	+	-	+	NA	+	+	+
-	-	+	-	-	-	+	-	-	-	+	+	NA	+	+	-
-	-	-	-	-	+	-	+	-	-	+	-	NA	+	+	+
+	-	-	-	-	-	-	-	-	-	-	-	NA	-	-	-
-	-	+	+	+	+	-	+	+	-	-	_	NA	+	-	+
+	-	-	+	-	-	-	-	-	+	-	-	NA	+	+	-
+	-	-	+	-	-	-	-	-	-	-	+	NA	-	+	+
-	-	+	+	-	+	+	+	-	+	-	-	NA	-	+	+
-	-	-	+	-	+	+	-	-	-	-	-	NA	-	+	-
-	-	-	-	-	-	-	-	-	-	-	-	NA	-	-	-
+	-	+	-	-	-	-	-	-	+	+	-	NA	+	-	+
-	-	-	-	-	-	-	-	+	-	-	-	NA	-	-	-
-	-	+	+	+	+	-	-	-	+	-	+	NA	-	+	-
-	-	-	-	-	-	-	-	-	-	-	-	NA	-	-	-
+	+	+	+	+	+	-	-	-	-	-	-	NA	-	-	+
-	-	-	-	-	-	+	-	-	-	-	-	NA	-	-	-
+	+	+	+	+	+	+	+	-	-	-	_	NA	-	-	-
+	-	+	+	+	+	+	-	-	-	-	-	NA	-	-	-
-	-	-	-	-	-	-	-	-	-	-	-	NA	-	-	+
-	-	-	-	+	-	+	-	-	-	-	-	NA	-	-	-
-	+	-	+	+	+	-	+	-	-	-	-	NA	-	-	+
Male	Male	Male	Female	Female	Female	Male	Female	Male	Male	Female	Male	Male	Female	Male	Male
Lor	Lor	Lor	Arab	Lor	Arab	Arab	Lor	Lor	Lor	Arab	Arab	Arab	Arab	Arab	Arab
+	+	+	+	+	+	+	+	-	-	NA	+	-	+	+	+
16Y	7Y	13Y	18Y	16Y	12Y	9Y	11Y	7Y	14Y	NA	5Y	ND	12Y	14Y	10Y

**TABLE 2:** In silico pathogenicity analysis of the detected variants. WES: Whole Exome Sequencing, NF: not found, D: deleterious, N: neutral, NA: not available.

Proband	Gene	Variant GRCh37 chr location CDS protein change	Consequence	GenomAD (AF)	Predict SNP2	Polyphen2	Mutation Taster	CADD score	REVEL score	ClinVar	Varsome (ACMG classification)	Method of detection
			Zygosity									
P1	IDUA	chr4:994486:G>A	Intronic/Splice site	NF	89%D	NA	Deleterious	33	NA	NA	Pathogenic (PVS1, PM2, PP4)	WES
		NM_000203.5:c.385 + 1G>A	Homozygote									
		-										
P2	IDS	chrX:148579644:GT>G NM_000202.8:c.701del	Frameshift	NF	NA	Probably damaging	Deleterious	NA	NA	NA	Pathogenic (PVS1, PM2, PP4)	WES
		p.Tyr234SerfsTer46	Hemizygote			Score:1						
P3	IDS	chrX:148585021:T>C	Intronic/Splice site	NF	64%N	NA	NA	34	NA	NA	Pathogenic (PVS1, PM2, PP4)	WES
		NM_000202.8:c.241-2A>G	Hemizygote									
		-										
P4	IDS	chrX:148564524:G>C	Missense	NF	87% D	Probably damaging	Deleterious	24.5	0.761	Likely pathogenic	Likely pathogenic (PM1, PM5, PP3, PP5, PM2, PP4)	WES
		NM_000202.8:c.1406C>G p.Pro469Arg	Hemizygote			Score: 0.994						
P5	IDS	chrX:148564635:C>T	Missense	NF	87% D	Probably damaging	Deleterious	27.2	0.939	NA	Likely pathogenic (PP3, PM2, PP2, PP5, PP4)	Sanger sequencing
		NM_000202.8:c.1295G>A p.Cys432Tyr	Hemizygote			Score:0.997						
P6	IDS	chrX:148578049:T>C	Intronic/Splice site	NF	87% D	NA	Deleterious	35	NA	NA	Pathogenic (PVS1, PM2, PP4)	Sanger sequencing
		NM_000202.8:c.709-2A>G	Hemizygote									
		-										
P7	IDS	chrX:148582529:C>G	Missense	NF	82%D	Probably damaging	Deleterious	28.2	0.857	NA	Likely pathogenic (PM1, PM5, PP3, PM2, PP4)	Sanger sequencing
		NM_000202.8:c.458G>C p.Trp153Ser	Hemizygote			Score:1						
P8	IDS	chrX:148571931:A>C	Missense	NF	63%N	Probably damaging	Deleterious	24.9	0.896	NA	Likely pathogenic (PP3, PM1, PM2, PP4)	Sanger sequencing
		NM_000202.8:c.920T>G p.Leu307Trp	Hemizygote			Score:0.996						
P9	SGSH	chr17:78190931:A>G	Missense	NF	87% D	Probably damaging	Deleterious	26.1	0.786	NA	Likely pathogenic (PP3, PM2, PP2, PP4)	WES
		NM_000199.3:c.149T>C p.Leu50Pro	Homozygote			Score:1						
P10	SGSH	chr17:78184631:G>A	Missense	0.00001425	87% D	Probably damaging	Deleterious	29.9	0.876	Pathogenic/Likely pathogenic	Pathogenic (PP5, PM5, PP3, PM1, PM2, PP4)	Sanger sequencing
		NM_000199.5:c.1129C>T p. Arg377Cys	Homozygote			Score:1						
P11	NAGLU	chr17:40690432:C>T	Nonsense	0.000003717	72%D	NA	Deleterious	36	0	Pathogenic	Pathogenic (PVS1, PP5, PM2, PP4)	WES
		NM_000263.4:c.607C>T p.Arg203Ter	Homozygote									
P12	NAGLU	chr17:40688549:G>C	Missense	0.000	89% N	Probably damaging	Benign	23.6	0.619	NA	VUS leaning toward likely pathoegnic (PP3, PM2, PP2, PP4)	WES
		NM_000263.4:c.259G>C p.Ala87Pro	Homozygote			Score:0.994						
P13	NAGLU	chr17:40688549:G>C	Missense	0.000	89% N	Probably damaging	Benign	23.6	0.619	NA	VUS leaning toward likely pathoegnic (PP3, PM2, PP2, PP4)	WES
		NM_000263.4:c.259G>C p.Ala87Pro	Homozygote			Score:0.994						
P14	NAGLU	chr17:40695468:C>T	Missense	0.000006257	87% D	Probably damaging	Deleterious	23.8	0.9	Pathogenic/Likely pathogenic	Likely pathogenic (PP5, PP3, PM5, PM2, PP2, PP4)	WES
		NM_000263.4:c.1444C>T p.Arg482Trp	Homozygote			Score:1						
P15	NAGLU	chr17:40696069:T>G	Missense	6.843e-7	87% D	Probably damaging	Deleterious	25.2	0.894	Pathogenic	Likely pathogenic (PP3, PP5, PM2, PP2, PP4)	WES
		NM_000263.4:c.2045T>G p.Leu682Arg	Homozygote			Score:1						
P16	NAGLU	chr17:40695468:C>T NM_000263.4:c.1444C>T	Missense	0.000006257	87% D	Probably damaging	Deleterious	23.8	0.9	Pathogenic/Likely pathogenic	Likely pathogenic (PP5, PP3, PM5, PM2, PP2, PP4)	WES
		p.Arg482Trp	Homozygote			Score:1						
P17	NAGLU	chr17:40690432:C>T NM_000263.4:c.607C>T	Nonsense	0.000003717	72%D	NA	Deleterious	36	0	Pathogenic	Pathogenic (PVS1, PP5, PM2, PP4)	WES
		p.Arg203Ter	Homozygote									
P18	NAGLU	chr17:40690432:C>T	Nonsense	0.000003717	72%D	NA	Deleterious	36	0	Pathogenic	Pathogenic (PVS1, PP5, PM2, PP4)	Sanger sequencing
		NM_000263.4:c.607C>T p.Arg203Ter	Homozygote									
P19	NAGLU	chr17:40693129:A>G	Missense	0.000003181	87% D	Probably damaging	Deleterious	24.3	0.888	Pathogenic	Pathogenic (PP5, PM5, PP3, PM1, PM2, PP4)	Sanger sequencing
		NM_000263.4:c.926A>G p.Tyr309Cys	Homozygote			Score:1						
P20	NAGLU	chr17:40696069:T>G	Missense	6.843e-7	87% D	Probably damaging	Deleterious	25.2	0.894	Pathogenic	Pathogenic (PP3, PP5, PM2, PP2, PP4)	Sanger sequencing
		NM_000263.4:c.2045T>G p.Leu682Arg	Homozygote			Score:1						
P21	NAGLU	chr17:40688549:G>C	Missense	0.000	89% N	Probably damaging	Benign	23.6	0.619	NA	VUS (PP3, PM2, PP2, PP4)	Sanger sequencing
		NM_000263.4:c.259G>C p.Ala87Pro	Homozygote			Score:0.994						
P22	HGSNAT	chr8:43014188:G>A	Intronic/Splice site	NF	61%D	NA	Deleterious	34	NA	Pathogenic	Pathogenic (PVS1, PP5, PM2, PP4)	WES
		NM_152419.3:c.493 + 1G>A	Homozygote									
		-										
P23	HGSNAT	chr8:43002207:G>A	Intronic/Splice site	0.00002522	61%D	NA	Deleterious	32	NA	Pathogenic	Pathogenic (PVS1, PP5, PM2, PP4)	WES
		NM_152419.3:c.234 + 1G>A	Homozygote									
		-										
P24	GNS	chr12:65113952:T>A	Missense	NF	87% D	Probably damaging	Deleterious	32	0.929	NA	VUS (PP3, PM2, PP4)	WES
		NM_002076.4:c.1430A>T p.Glu477Val	Homozygote			Score:1						
P25	GALNS	chr16:88891195:A>AGTTG	Frameshift	NF	NA	NA	Deleterious	NA	NA	NA	Likely pathogenic (PVS1, PM2, PP4)	WES
		NM_000512.5:c.1218_1221dup p.Ser408GlnfsTer11	Homozygote									
P26	GALNS	chr16:88909203:G>A	Missense	NF	87% D	Probably damaging	Deleterious	27.1	0.951	Conflicting	Likely pathogenic (PP3, PM1, PM2, PP5, PP4)	WES
		NM_000512.5:c.155C>T p.Pro.52Leu	Homozygote			Score:1						
P27	GALNS	chr16:88891195:A>AGTTG	Frameshift	NF	NA	NA	Deleterious	NA	NA	NA	Pathogenic (PVS1, PM2, PP4)	Sanger sequencing
		NM_000512.5:c.1218_1221dup p.Ser408GlnfsTer11	Homozygote									
P28	ARSB	chr5:78264850:G>A	Nonsense	0.00001735	77%D	NA	Deleterious	42	0	Pathogenic	Pathogenic (PVS1, PP5, PM2, PP4)	WES
		NM_000046.5:c.478C>T p.Arg160Ter	Homozygote									
P29	ARSB	chr5:78280791:G>T	Nonsense	7.453e-7	NA	NA	Deleterious	39	0	Likely pathogenic	Pathogenic (PVS1, PM2, PP5, PP4)	Sanger sequencing
		NM_000046.5:c.281C>A p.Ser94Ter	Homozygote									
P30	ARSB	chr5:78280791:G>T	Nonsense	7.453e-7	NA	NA	Deleterious	39	0	Likely pathogenic	Pathogenic (PVS1, PM2, PP5, PP4)	Sanger sequencing
		NM_000046.5:c.281C>A p.Ser94Ter	Homozygote									
P31	ARSB	chr5:78280791:G>T	Nonsense	7.453e-7	NA	NA	Deleterious	39	0	Likely pathogenic	Pathogenic (PVS1, PM2, PP5, PP4)	Sanger sequencing
		NM_000046.5:c.281C>A p.Ser94Ter	Homozygote									
P32	ARSB	chr5:78251263:G>C	Nonsense	0.000001368	81%D	NA	Deleterious	35	NA	Pathogenic	Pathogenic (PVS1, PP5, PM2, PP4)	Sanger sequencing
		NM_000046.5:c.753C>G p.Tyr251Ter	Homozygote									

### 3.1 IDS

**FIGURE 2:**
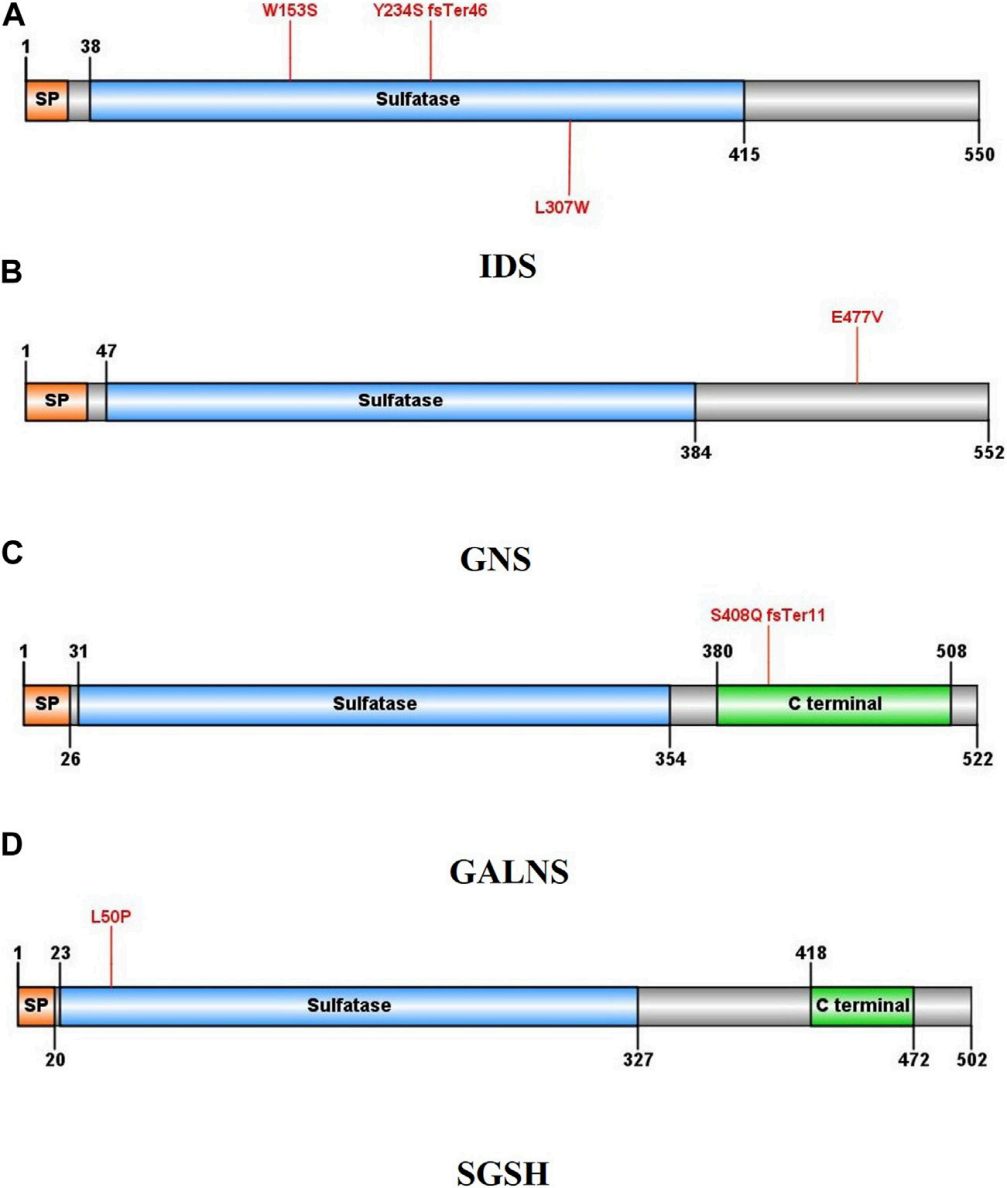
Schematic illustration of the protein structures in which at least one new variant was identified: **(A)** Iduronate-2-sulfatase, **(B)** N-acetylglucosamine-6-sulfatase, **(C)** galactosamine-6-sulfate sulfatase, **(D)** N-sulfoglucosamine sulfohydrolase. Mutated residues are indicated by red lines.

**FIGURE 3:**
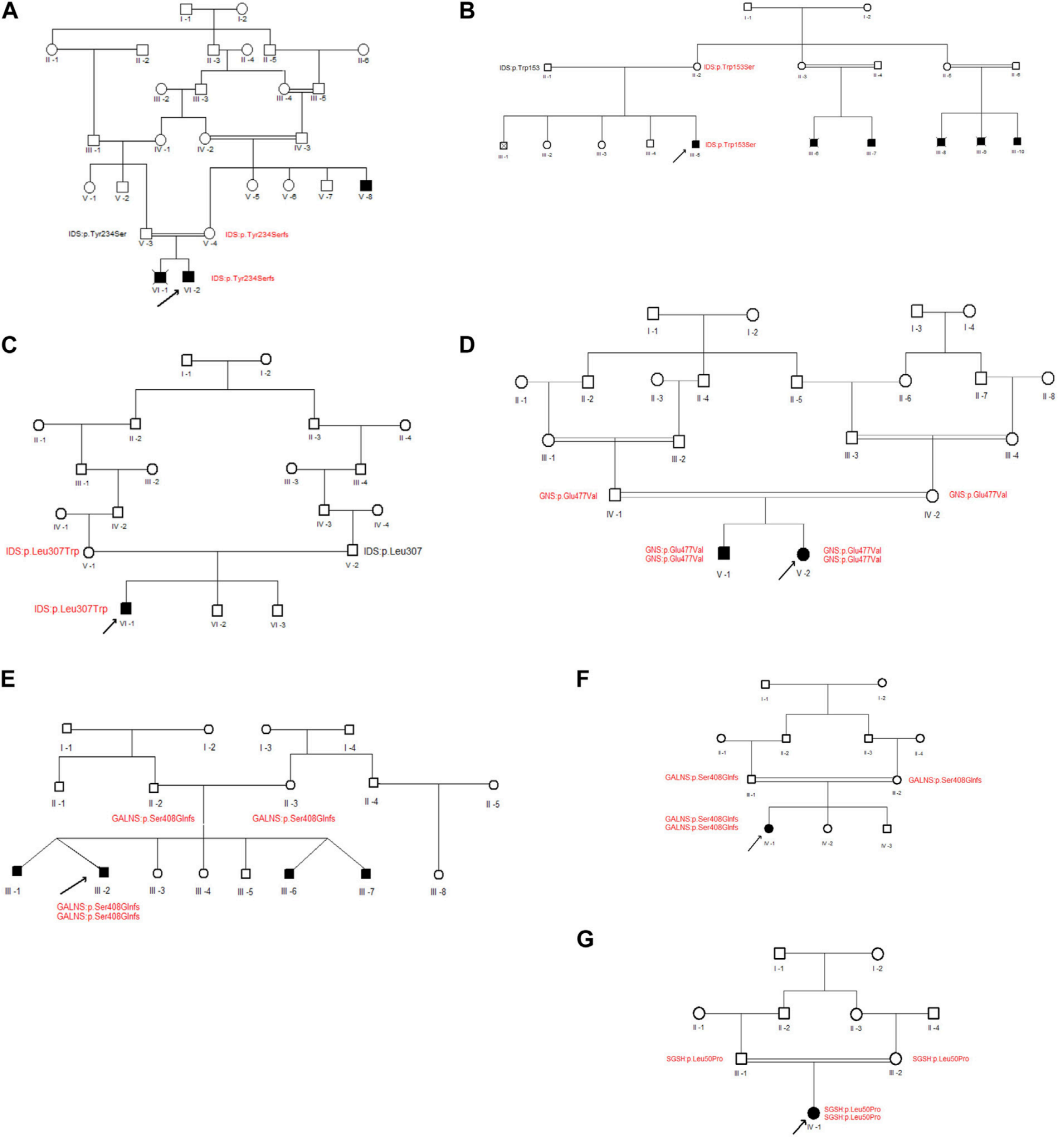
Pedigrees of families with new variants. **(A–G)** represent the family pedigrees of P2, P7, P8, P9, P24, P25 and P27 cases, respectively. Squares and circles denote males and females, respectively. Filled symbols represent affected individuals, and consanguinity is indicated by double marriage lines. The normal and mutated protein locations of participating individuals have been written beneath each individual in black and red, respectively. The proband is indicated by an arrow.

Three different variants were detected in the Iduronate-2-sulfatase (*IDS*) gene in P2, P7, and P8. As shown in Figure 2, all of the variants were found within the sulfatase domain of the protein, which is crucial for the enzyme’s activity. In the case of P2, genetic screening identified a single nucleotide deletion (NM_000202.8, c.701del, p.Tyr234Ser) in exon 5. Additionally, two novel missense variants, (NM_000202.8, c.458G>C, p.Trp153Ser (exon 4)) and (NM_000202.8, c.920T>G, p.Leu307Trp (exon 7)), were detected in subjects P7 and P8, respectively. All three variants were found in a hemizygous state in the probands, while being heterozygous in their mother and wild type in their father. According to the pedigrees of the families (Figure 3), the other patients in these three families were also males, confirming the type of disease as mucopolysaccharidosis type II.

### 3.2 GNS

In case P24, a missense variant was detected in the N-acetylglucosamine-6-sulfatase (*GNS*) gene (NM_002076.4, c.1430A>T, p.Glu477Val). This variant was located in exon 13 and similar to the variants in the *IDS* gene, it affects the sulfatase domain of the protein. As shown in Figure 3, The proband was homozygous for the mentioned variant, and both of her parents were heterozygous. After performing Sanger sequencing on the proband’s brother, who was also suspected of having mucopolysaccharidosis, it was revealed that he also has this variant in a homozygous form.

### 3.3 GALNS

In patients P25 and P27, a novel duplication variant was identified in exon 11 of the galactosamine-6-sulfate sulfatase (*GALNS*) gene (NM_000512.4, c.1218_1221dup, p.Ser408GlnfsTer11). In both cases, the probands inherited the variant homozygously from their heterozygous parents. Interestingly, in case P25, the patient also had an identical twin with him, along with two other identical twin brothers who also had mucopolysaccharidosis type IVA.

### 3.4 SGSH

A missense variant was found in exon 2 of the N-sulfoglucosamine sulfohydrolase (*SGSH*) gene in case P9 (NM_000199.3, c.149T>C, p.Leu50Pro). This variant also affects the sulfatase domain of the protein, potentially interfering with its activity. Here, the proband was the only affected individual in the family and had received the variant in a homozygous form from her heterozygous parents.

## 4 Discussion

In the present study, a total of 32 cases with a primary diagnosis of mucopolysaccharidosis from separate families underwent genetic analysis. In some families, there was only one patient, while in others, there were additional patients besides the proband, clearly indicating the hereditary nature of the disease. Among all the families, MPS I was reported in only one family, and the affected individual was characterized as having the MPS Ih/s subtype. Genetic analysis revealed a homozygous splice site variant located in intron 3 of the *IDUA* gene (c.385 + 1G>A) in the proband. This variant had previously been detected in the European population and was shown to cause the loss of a neutral donor splice site that is conserved among vertebrate species (Bertola et al., 2011).

Probands in seven families were classified as MPS II, and we identified four previously reported and three novel hemizygous variants in the *IDS* gene. X-linked inheritance was apparent in the pedigrees and all of the patients were male. Among these variants, an intronic splice variant (c.241-2A>G) was detected, which can lead to alternative splicing and the creation of two mutant transcripts (Alves et al., 2006). Another splice site variant (c.709-2A>G) was detected, in which a nucleotide transition leads to a 3′ splice site alteration in intron 5 of the *IDS* gene (Lissens et al., 1997). This alteration may result in the production of an incorrect transcript or a premature stop codon, leading to the absence of a functional protein. Further investigation and functional assays are needed to determine the exact mechanism of this vatiant. Other previously reported alterations in MPS II patients were two missense variants, including c.1406C>G and c.1295G>A (Karsten et al., 1998; Pollard et al., 2013). Additionally, two previously unreported missense variants were identified in this study. In one of these variants (c.458G>C, p.Trp153Ser), a hydrophobic, nonpolar, aromatic, and large amino acid is replaced with a neutral, polar, and small amino acid. Hence, factors such as hydrophobicity, size and polarity exchange may affect the final protein folding. Two other pathogenic variants in the same amino acid have been previously reported, in which tryptophan is converted to arginine and leucine, respectively (Froissart et al., 2007; Zhang et al., 2019). In another missense variant (c.920T>G, p.Leu307Trp), an aliphatic amino acid is changed to an aromatic amino acid. This change allows the amino acid to donate hydrogene, unlike its previous form. In addition to the missense variants, one frameshift variant was also found in the *IDS* gene (c.701del, Tyr234Ser), which likely results in the production of incomplete and non-functional protein.

Sanfilippo syndrome, the most frequent and heterogeneous type of mucopolysaccharidoses (van de Kamp et al., 1981), was observed in the families with different subtypes ranging from MPS IIIA to MPS IIID. In two of the families, we identified two distinct homozygous missense variants in the *SGSH* gene. These variants included one previously reported variant (c.1129C>T) and one newly discovered variant (c.149T>C), both of which were found to be responsible for causing MPS ⅢA. The c.1129C>T variant, which was initially identified by (Di Natale et al., 1998) leads to a severe form of the disease because position 377 at the C-terminal is crucial for sulfamidase function (Di Natale et al., 1998). Our studied patient had a severe form of the disease, consistent with the research conducted by Di Natale et al. The c.149T>C variant, p.Leu50Pro, involves the substitution of a hydrophobic and large amino acid with a neutral and small amino acid. This change in the size and nature of amino acids at position 50 may affect the protein’s interactions with other molecules and residues, leading to improper protein folding. Among the individuals we studied, the highest frequency of Sanfilippo patients was associated with the subtype of MPS ⅢB. In this subtype, we observed five different variants in the *NAGLU* gene. Among these variants, we found a previously reported nonsense variant (c.607C>T, p.Arg203ter) and a missense variant (c.2045T>G, p.Leu682Arg) in three and two families, respectively. Schmidtchen et al. (1998) first reported these variants and demonstrated the presence of these variants along with 8 other variants in the fibroblast cell line of patients with MPS ⅢB. They also validated the reduction of enzyme activity in Chinese hamster ovary cells transfected with a mutagenized vector containing the *NAGLU* missense variants. Furthermore, they found excessive molecular heterogeneity in the *NAGLU* gene and suggested a role for the amino or carboxyl end of α-N-acetylglucosaminidase in the transport or function of the enzyme (Schmidtchen et al., 1998). In three families, we identified one homozygous missense variant (c.259G>C, p.Ala87Pro) in the *NAGLU* gene. This variant is similar to the aforementioned variants in terms of its location in the N-terminal domain of the protein. The substitution of alanine with proline converts a hydrophobic amino acid to a neutral one. As a result, the hydrophobic intra and intermolecular interactions that are dependent on this substitution lead to the complete loss of protein activity. Pollard et al. (2013) reported the c.259G>C substitution in a compound heterozygous patient, in combination with another variant (c.1949G>A, p.G650E) (Pollard et al., 2013). However, our patients showed novel homozygous alleles. In two of the families we observed a substitution (c.1444C>T) in the *NAGLU* gene, where a 5-methylcytosine in a highly mutable CpG dinucleotide position is converted to thymine as a result of deamination (Bunge et al., 1999). The last observed missense variant in the *NAGLU* gene in our study was a substitution (c.926A>G, p.Tyr309Cys) in a highly conserved amino acid. This variant does not affect the amount or stability of RNA, but it only influences the activity of the enzyme (Lee-Chen et al., 2002). We identified MPS ⅢC in two families, where we observed two different previously described homozygous splice site variants (c.493 + 1G>A, c.234 + 1G>A) in the *HGSNAT* gene (Fan et al., 2006; Hřebíček et al., 2006). In a comprehensive study, (Martins et al., 2019), described the evolutionary history of MPS ⅡIC by analyzing the clinical presentation, molecular defects, and haplotype context of patients from 22 countries (Martins et al., 2019). Remarkably, both patients in our study were from the Arab ethnic group, suggesting an African origin of these variants. In one of the families, we found the c.1430A>T missense variant in the *GNS* gene, which is responsible for MPS IIID. This variant was found for the first time in the current study. At the protein level, this variant results in the conversion of a hydrophilic, negatively charged and polar amino acid to a hydrophobic, uncharged and nonpolar amino acid. This change has the potential to generate misfolded proteins. In two families with individuals clinically diagnosed with MPS ⅣA, a novel homozygous frameshift variant was observed in the *GALNS* gene. This variant is caused by a duplication of CAAC at position 1,218 to 1,221 of the coding sequence (c.1218_1221dup); resulting in the introduction of a premature termination codon (PTC) 11 codons downstream of the duplication. Another MPS ⅣA patient was found to be homozygous for a missense variant (c.155C>T) that had been previously identified in the Indian population. This variant is likely to prevent the formation of homodimers of the GALNS protein, as reported by (Bidchol et al., 2014). Additionally, two different nonsense variants were identified in the *ARSB* gene, which is mutated in MPS VI. These variants are c.478C>T and c.281C>A, resulting in the conversion of Arg160 and Ser94 to premature stop codons and the creation of a non-functional truncated protein (Voskoboeva et al., 1994). The c.478C>T variant is located in a CpG dinucleotide, which is considered a mutation hotspot in the *ARSB* gene, as noted by (Zapała et al., 2020). It is also one of the most frequently reported pathogenic and likely pathogenic variants in ARSB, as stated by (Tomanin et al., 2018). The c.281C>A variant was found to have a high frequency in Arab patients, as reported by (Aminzadeh et al., 2019). Its presence in 4 out of 5 families of Arab ethnicity suggests that this variant may be the most common variant among Iranian Arab patients with MPS VI. Furthermore, among the four families with the same variant, one family experienced neonatal death, while the individuals in the other cases were over 10 years old. This may demonstrate the clinical heterogeneity of MPS VI.

In mucopolysaccharidoses, as was the case in the current study, there can be clinical symptom overlap between different types. In some cases, relying solely on clinical symptoms and biochemical tests for an accurate diagnosis of Mucopolysaccharidosis type can lead to errors. However, with the assistance of genetic counseling and genetic tests, an accurate diagnosis of Mucopolysaccharidosis type in patients can be achieved. For instance, in the case of MPS type 2, which has X-linked inheritance, a definitive diagnosis can be made by combining genetic counseling, identifying the disease more frequently in males than females, and detecting mutations in the IDS gene.

This research is unique due to the prevalence of specific ethnic groups and the practice of consanguineous marriage in them. Considering that all types of mucopolysaccharidosis, except type II, have autosomal recessive inheritance, the high rate of inbreeding leads to the emergence and manifestation of potentially pathogenic variants in populations with high levels of inbreeding. Therefore, further genetic investigations and studies, particularly those utilizing exome or genome sequencing, are strongly recommended in inbred populations.

There are several caveats that should be noted regarding the present study. Firstly, in most cases, the other patients in the family, apart from the proband, were not sequenced. This was either due to their unavailability or their refusal to participate in the research. Secondly, the current study does not include functional assays related to the novel detected variants, and our final criteria for determining the pathogenicity of variants were based on ACMG classification. Therefore, future research is needed to validate the pathogenicity and better understand the mechanism and biological effects of the identified new variants. These studies should include more *in silico* investigations, such as 3D protein structure analysis, as well as *in-vitro* and *in-vivo* functional assays.

## 5 Conclusion

Genetic screenings can provide significant benefits in managing and preventing diseases within families, as well as alleviating clinical symptoms in new cases (Kubaski et al., 2020). In this study, we focused on whole exome sequencing and Sanger sequencing to examine genetic variants in patients with mucopolysaccharidosis from the Khuzestan province in Southwest Iran. We detected six novel variants and confirmed these findings through segregation analysis in family members. Our *in silico* analysis suggests that these novel variants may be disease-causing variants. The present findings contribute to expanding the spectrum of pathogenic variants associated with mucopolysaccharidosis and can facilitate rapid diagnosis.

## Data Availability

The datasets presented in this article cannot be publicly shared due to privacy restrictions. Requests to access the datasets should be directed to the corresponding authors.
